# Italian Children’s Well-Being after Lockdown: Predictors of Psychopathological Symptoms in Times of COVID-19

**DOI:** 10.3390/ijerph182111429

**Published:** 2021-10-30

**Authors:** Marcella Caputi, Barbara Forresi, Ludovica Giani, Giovanni Michelini, Simona Scaini

**Affiliations:** 1 Department of Life Sciences, University of Trieste, Via E. Weiss 21, 34128 Trieste, Italy; 2 Child and Youth Lab, Sigmund Freud University of Milan, Via Ripa di Porta Ticinese 77, 20143 Milan, Italy; b.forresi@milano-sfu.it (B.F.); giani.phd@milano-sfu.it (L.G.); g.michelini@milano-sfu.it (G.M.); s.scaini@milano-sfu.it (S.S.)

**Keywords:** COVID-19, resilience, adverse childhood experiences, temperament, flooding

## Abstract

The first Italian lockdown imposed to fight the spread of COVID-19 caused important disruptions in families’ everyday lives. The main aim of this research was to investigate the predictors of psychopathology in children aged 5–10 years, immediately after the national 2-month lockdown. A total of 158 Italian parents (148 mothers, 10 fathers, mean age = 41 years) were recruited and asked to complete an online research concerning their 158 children (76 boys, mean age = 7.4 years). Parents completed questionnaires on parent–child conflict, resilience, temperament, behavior, and previous adverse childhood experiences. Hierarchical regressions showed that children’s psychopathology was predicted by low child resilience, high novelty seeking and harm avoidance, adverse experiences, and high flooding levels. Moreover, girls exposed to adverse experiences appeared more vulnerable to psychopathology. The recruitment of a convenience sample, the small sample size, and the cross-sectional design of our study limit the generalizability and interpretation of the present findings. Nonetheless, this research extends our knowledge of children’s functioning in such an exceptional period. Shedding light on predictors of children’s psychopathology following prolonged quarantine can indeed guide effective psychological interventions now and in future similar situations.

## 1. Introduction

All countries in the world have been fighting COVID-19 since December 2019. Italy has been one of the most affected European countries and was the first to apply strict measures trying to contain the outbreak. In March 2020, the Italian Prime Minister, with a Decree called “I stay at home”, declared the first national lockdown confining millions of people to their homes and banning any form of social aggregation. This entailed severe travel restrictions and immediate shutdown of schools and most workplaces. For two months, people could not leave their home except for essential needs.

Whenever possible, people began to work remotely from their houses, but many stopped working completely or lost their jobs. The first international epidemiological screenings soon highlighted worrying levels of depressive symptoms, anxiety symptoms, and stress among the general population [1,2]. The effects on families with preschoolers and school-aged children are particularly interesting, as parents suddenly had to turn into teachers, friends, and grandparents at the same time; while houses also became classrooms, workplaces, and play spaces. Since the start of the COVID-19 emergency, many parents have been working remotely. Under these conditions, the risk of burnout is very high [3,4], and so is the level of parental stress children are exposed to. When caregivers are stressed out, children do not receive the help they need to regulate their emotions and may experience overwhelming tension. In a vicious cycle, children may assimilate parents’ stress and negativity, which leads to behaviors that exacerbate these feelings, thereby putting at risk the whole family’s wellbeing [5]. However, the family microsystem might react to external threats enacting positive interactions and ensuring mutual support, manifesting resilience, and growing stronger [5,6].

Publications on previous epidemics requiring a certain amount of quarantine reported psychological distress in the short term [7,8], and post-traumatic growth and resilience in the long term [6,9,10]. As far as COVID-19 is concerned, literature so far indicates sleep problems and psychological difficulties (e.g., fear, clinging, inattention, and irritability) among children [3,11,12], and sleep problems, psychological distress, and internalizing symptoms among parents [1,3,13].

Nonetheless, the role played by individual and relational factors in explaining children’s behavior after prolonged quarantine still needs to be unfolded.

### 1.1. Conceptual Framework

Children’s resilience in the face of prolonged stress results from a combination of protective factors operating at individual and family/community levels. Although a prolonged activation of the stress system is toxic, caring adults providing responsive interactions can help children to transform toxic stress into tolerable stress [14].

According to the diathesis–stress model, disorders result from an interaction between a predisposition vulnerability and experienced stress [15]. Relations among family wellbeing, caregiver’s wellbeing, and children’s adjustment are not unidirectional, and risk factors operate within a mutually reinforcing system. Pre-existing vulnerabilities within the family increase susceptibility to maladjustment, with children experiencing multiple risk factors being more prone to psychological disorders [16,17].

### 1.2. The Present Study

In the present research, we evaluated the effect of several potential risk factors for the development of psychopathological symptomatology in quarantined Italian children during the COVID-19 outbreak. Since specific temperamental patterns are risk factors for later externalizing and internalizing disorders [18,19,20], we assessed four dimensions described by Cloninger [21]: novelty seeking, harm avoidance, reward dependence, and persistence.

We also measured adverse childhood experiences that occurred before quarantine, as accumulation of risks facilitates later mental problems [22,23].

Parent–child relationships are also likely to play a role in children’s adjustment, with an unexplored aspect represented by flooding, i.e., the extent to which a family member’s emotion is experienced as overpowering and distressing [24]. Flooding may be experienced by parents when children unexpectedly express negative affect and might lead parents to employ non-effective parenting, offering the quickest escape from a child’s negative affect but amplifying it in the long term [25,26]. It is usually associated with child’s externalizing behavior and poor satisfaction with the parenting role [25,26,27].

People (re)adapt very differently in response to adversities [28,29]. Resilient individuals have good mental health despite exposure to serious stress [28]. Resilience changes over time and is influenced by personal strengths and by resources provided in a facilitative environment [30]. Indeed, global resilience can be seen as the result of individual, family-related, and community-related resilience factors [28]. In the case of children and adolescents, family factors may bolster internal coping resources more than any other factor [31]. Even if all humans have a certain degree of resilience, this is highly dependent on the presence and accessibility to different kind of resources. Therefore, we assessed children’s and parents’ resilience, as both might protect children from developing psychopathological symptoms in a very stressful time.

Finally, we took into account the potential role played by specific COVID-19 related risk factors as emerged by recent literature. For example, Zijlmans and colleagues [32] showed that unemployment due to COVID-19 and having friends/relatives infected by the virus predict internalizing symptoms in Dutch children and adolescents.

We expected a significant role of children’s (temperament, resilience, and previous adverse experiences), parental (resilience and flooding), and COVID-19 related factors in predicting children’s psychological difficulties.

## 2. Materials and Methods

### 2.1. Participants

A sample of 158 Italian parents was recruited online (148 mothers and 10 fathers, mean age 41 years, SD = 5.3 years). Parent completed questionnaires for themselves and for their child. Therefore, we also gathered data on 158 children between 5 and 10 years of age (48% boys, mean age = 7.4 years; SD = 1.8 years).

### 2.2. Procedure

Parents were asked to complete the project questionnaires through an online survey (via Google Forms). This was advertised on social media by the office of marketing and communications of Sigmund Freud University and made available from 5 June to 30 June 2020 (in the immediate aftermath of the first national lockdown). Information regarding the study and confidentiality issues were indicated on the project landing page. Inclusion criteria were living in Italy and having a child aged between 5 and 10 years old. Exclusion criteria were not living in Italy and not having a child aged between 5 and 10 years old.

### 2.3. Ethical Statement

Ethics approval was obtained on 2 June 2020 by the Ethics Committee of Sigmund Freud University (Milan, Italy) in accordance with the ethical standards of the Declaration of Helsinki. Informed consent was obtained from each participant at the beginning of the survey.

### 2.4. Measures

Parents provided information on theirs and their child age and gender, their education and occupation, region of residence, and completed an assessment protocol that included the following measures and required about 20 min. 

The CYW Adverse Childhood Experiences Questionnaire CYW ACE-Q [33]—Child version. This is a clinical screening tool that calculates cumulative exposure to Adverse Childhood Experiences (ACEs) in children aged 0–12 years. Parents were asked to report how many experience types apply to their child. It is composed of 17 items: 10 assessing exposure to the original ACEs and 7 to additional early life stressors. Translation into Italian followed published guidelines, including the use of independent back translation.

The Child and Youth Resilience Measure—Person Most Knowledgeable version CYRM-PMK [34]. The CYRM-PMK measures resources available to children that may bolster their resilience. It is composed of 17 items with the following answer options: yes, no, or sometimes. High scores indicate high resilience skills. Translation into Italian followed published guidelines, including the use of independent back translation.

The Adult Resilience Measure—ARM-R [35]. The ARM-R is a self-report measure of social-ecological resilience, widely used by researchers worldwide. It is composed of 17 items with the following answer options: yes, no, or sometimes. High scores indicate high resilience.

Junior Temperament and Character Inventory (JTCI) [36,37]. Adapted from the Temperament and Character Inventory, developed for adults and by Cloninger in 1994, this instrument assesses temperament (novelty seeking, harm avoidance, reward dependence, persistence) and character (self-directedness, cooperativeness, self-transcendence) in children and adolescents. Only temperament items were administered in the present research.

The Strengths and Difficulties Questionnaire [38,39]. This questionnaire assesses mental health problems and psychological adjustment in children aged 3–16 years. It consists of 25 items divided in five subscales: emotional symptoms, conduct problems, hyperactivity-inattention, peer problems, and prosocial behavior. Each item is scored on a 3-point scale (0 = not true, 1 = somewhat true, 2 = certainly true). In the present research, only the total difficulties score (obtained summing all the subscales but prosocial behavior) was used in the analyses.

The Parental Flooding Scale [27]. This is a 15-item measure designed to assess the degree to which parents experience their children’s negative affect expressed during parent–child conflicts as unpredictable, overwhelming, and disorganizing. Items are rated from 1 = almost always to 5 = never, with high scores indicating low flooding. Translation into Italian followed published guidelines, including the use of independent back translation.

COVID-19 risk index (created ad hoc). We collected the following information (each question had a yes/no format, and every positive answer was given 1-point, total score = 0–5): having contracted COVID-19 virus personally or within the family; bereavements within the family due to COVID-19 virus; forced isolation/quarantine due to COVID-19 virus; living in a small house with no outdoor spaces during lockdown; unemployment due to COVID-19.

### 2.5. Data Analysis Plan

We conducted descriptive statistics, *t*-tests, and correlations. Then we ran regression analyses to evaluate the role of child and parent characteristics in predicting children’s psychopathological symptoms. Regressions were also run separated by gender in order to detect potential distinct patterns for boys and girls. Power calculations for *t*-tests, correlations, and regressions were performed at a level of 0.90. Calculations for detecting a medium effect size yielded an estimated sample size of 132, 111, 140, and 65 subjects for *t*-tests, correlations, whole-sample regressions, and gender-split regressions, respectively. The survey was discontinued one week after reaching the 140th participant.

## 3. Results

With regard to parental occupation, 41.8% of the respondents were full-time employees, 32.9% were freelancers, 13.4% were part-time employees, 6.9% were full-time parents, 4.4% were in managerial positions, 0.6% were students. With regard to parental education, 76.2% of the respondents had a University degree, 20% had the equivalent of A-levels (i.e., high school diploma), and 3.8% had the equivalent of General Certificates of Secondary Education (GCSEs). In terms of geographical distribution, 86% of the families were from the North of Italy, 8.9% from the Center, 5.1% from the South. 

### 3.1. Preliminary Analyses

Independent-samples *t*-tests showed no significant gender differences in the study variables (see Table 1). Table 1 also shows descriptive statistics with mean values for each study variable. 

As far as psychopathological symptoms are concerned, after two months of quarantine, 79.1% of children reported an SDQ total difficulties score in the normal range, 8.9% of children reported a borderline score, while 12% of children had symptoms in the abnormal range.

Before commenting on the main results, we report the correlations among all the study variables as a prelude to our model testing. As shown in Table 2, children’s resilience shows a high positive correlation not only with parental resilience but also with low levels of parental flooding. Positive significant correlations were also found among children’s resilience, low levels of emotional and behavioral difficulties (SDQ), low levels of novelty seeking, and high levels of reward dependence. With regard to ACEs, a higher number of events was related to lower levels of resilience as well as to higher SDQ symptoms.

### 3.2. Main Analyses

We performed a hierarchical regression analysis to evaluate predictors of the SDQ total difficulties score. Child resilience was the only predictor in the first model, in the second we added the COVID-19 risk index, in the third the four temperamental dimensions of JTCI (novelty seeking, harm avoidance, reward dependence, and persistence), in the fourth we added the total number of ACEs, in the fifth we added parental flooding, and in the last one the parental resilience. These analyses were first conducted on the whole sample and then repeated separately for boys and girls.

The results of the regression analysis on the whole sample are shown in Table 3 (coefficients are presented as standardized beta). ANOVA confirmed that the fifth and final model explained 64% of the variance (*R*^2^ = 0.640, *F*(9, 148) = 29.29, *p* < 0.001). Reward dependence, persistence, exposure to COVID-19-related risk factors, and parental resilience did not predict SDQ total difficulties. Significant predictors were low child resilience, high novelty seeking and harm avoidance, ACEs, and high levels of parental flooding.

The same regression analyses performed separately in boys and girls are shown in Table 4 and Table 5. Both models were significant (*F*(9, 66) = 21.04, *p* < 0.001 for boys and *F*(9, 72) = 10.09, *p* < 0.001 for girls), but the explained variance was higher in the boys model (*R*^2^ = 0.742 boys vs. 0.558 girls).

SDQ total difficulties in boys were predicted by low child resilience, high novelty seeking, and high levels of parental flooding. Differently, SDQ total difficulties in girls were predicted by high novelty seeking and harm avoidance, high levels of parental flooding, and ACEs.

## 4. Discussion

The main aim of the present study was to explore which kind of factors explain psychopathological difficulties in children after the first Italian national lockdown due to COVID-19 (from March to May 2020). It is worth noticing that 21% of Italian children exposed to a prolonged quarantine reported scores in the borderline or abnormal range, with 12% reporting severe emotional and behavioral difficulties. Such scores, albeit concerning a relatively small portion of the sample, appear to be in line not only with the normative scores of the SDQ in Italian children [40], but also with the results of another study conducted in a sample of Chinese children during the COVID-19 pandemic [41], showing that 17.6% of students was suspected to have emotional/behavioral problems. Although these results do not necessarily imply a worrisome trend in the level of psychopathology after quarantine, the borderline/abnormal total difficulties score detected in 21% of our sample could be the signal of a generalized distress experienced by children that warrants to be monitored in long-term follow-ups. 

Overall, our results revealed that children’s psychopathological difficulties were significantly predicted by high levels of novelty seeking and harm avoidance, low child resilience skills, high negative emotions experienced by parents during parent–child conflicts (flooding), and adverse childhood experiences. Therefore, in line with past studies, two specific temperamental dimensions predicted emotional and behavioral difficulties in children after quarantine: propensity for approach and exploration (novelty seeking), which had been previously found in association with impulsive behaviors and risky activities, and propensity to withdraw and worry (harm avoidance), which is usually related to anticipatory anxiety [42]. 

In the present research, children’s psychopathological symptoms were also significantly predicted by parental flooding. A forced and prolonged quarantine implied much more time spent together, that probably increased the number of parent–child conflictual situations. In stressful conditions, parents are likely to interact with their children less appropriately. Inappropriate parental reactions, that entail responding with maladaptive and less effective strategies to quickly escape from aversive experiences (i.e., responding to a screaming child by yelling back or giving in to their demands), could depend on individual factors and acquired skills (including resilience), but also on parent–child exchanges. This hypothesis was supported by our correlations, in that high levels of flooding were related to low resilience skills in parents and to high novelty seeking traits in children.

Another significant predictor of children’s psychopathological symptoms after COVID-19 quarantine in the present study was represented by adverse childhood experiences. Our results confirmed previous studies showing that outburst to high levels of environmental influences can increase the risk for psychopathology. Adverse childhood experiences have indeed been linked to later emotional and behavioral problems [43,44,45] and increased risk for poly-victimization [46]. Overall, then, our results supported previous findings showing a dose–response relationship between cumulative adverse childhood experiences and psychopathological outcomes [45,47,48].

The last significant predictor of children’s psychopathological symptoms in our sample was child resilience. Specifically, low resilience skills predicted high SDQ total difficulties scores. This is in line with the compensatory model of resilience that underscores the role of resilience in promoting a good psychological functioning and hindering the negative impact of adversities [49]. Parental resilience, on the other hand, did not predict child psychopathological symptoms. Family resilience has been previously found to promote flourishing in children, even amid adversity [50,51]. Nonetheless, it is a distal factor and therefore its influence on the outcome is probably lower compared to proximal factors. Overall, children’s resilience should be then especially increased when planning interventions aimed at tackling children’s problematic outcomes. Unexpectedly, no association emerged between COVID-19 risk factors and children’s psychopathological difficulties. This could be due to the fact that our sample was not significantly affected by a high number of the risk factors that we chose to investigate: 67% of participants reported the exposure to none of them.

Interestingly, in our study, both in boys and in girls, higher levels of psychopathological symptoms were predicted by higher novelty seeking and parental flooding scores. However, only among boys, lower resilience skills played a significant predictive role in psychopathology; whereas only among girls, higher harm avoidance scores and adverse childhood experiences predicted psychopathological symptoms. Thus, predictors of psychopathological symptoms were different according to gender. On one hand, low levels of resilience skills seem to put boys at risk for developing difficulties, which seems coherent with a protective mechanism recently found among Italian boys (and not girls) exposed to a traumatic event. In the study by Stratta and colleagues [52], boys showed higher resilience skills and problem-focused strategies in the face of a negative event. On the other hand, girls seem to be more influenced by stressful events, which is in accordance with previously reported data showing a stronger association between stressful events and psychopathological problems in girls compared to boys. Indeed, girls could be more sensitive to the effects of stressful life events during the transition from childhood to adolescence [53] and more predisposed to rumination, leading to emphasize the role and the effect of adverse experiences [54,55]. Moreover, girls more prone to withdraw and worry (i.e., with higher harm avoidance scores) seem to be at a higher risk for developing difficulties. Such temperamental trait could be further increased by the experience of adversities in the early years of life.

## 5. Limitations and Conclusions

Some limitations of the present study must be acknowledged. The first one concerns the sample and weakens the generalizability of our results. Indeed, we recruited a convenience sample, which *per se* has a high degree of bias. Moreover, in the aftermath of the lockdown period, few parents were available to complete our questionnaires and were mostly from Northern Italy and with overall high education level. Therefore, we are not sure that our sample is representative of Italian parents of children aged 5–10 years old. The second limitation is represented by the cross-sectional design of the study, which does not allow to know the level of children’s symptomatology pre-COVID-19. The third limitation is that all measures were completed by parents, raising the issue of shared method variance. In addition, most of respondents were mothers. Therefore, answers to our questionnaires actually provide more mothers’, than fathers’, points of view.

Nonetheless, the present research extends our knowledge of children’s functioning in such an exceptional period. Overall, both children’s and parental characteristics emerged as risk/protective factors for the development of psychopathological symptoms in school-age children, immediately after the lockdown period, indicating the importance of considering both biological and environmental factors. Significant predictors of SDQ total difficulties, in the present investigation, were child resilience, the temperamental dimensions of novelty seeking and harm avoidance, adverse childhood experiences, and parental flooding. The quarantine imposed during the first national lockdown determined a massive consumption of personal resources, both in parents and in their children, to cope with a new everyday routine and worries about future. Therefore, public health efforts should be oriented especially towards enhancing child resilience and supporting effective parenting, given that these two variables are the only modifiable factors that emerged among the significant predictors of children’s psychopathology.

We believe that understanding parents’ and children’s reactions and emotions, and identifying risk and protective factors of psychopathology, is essential to properly ad-dress families’ needs to tailor present and future intervention programs in the unlucky event of another lockdown [4].

## Figures and Tables

**Table 1:** Descriptive statistics and *t*-tests for gender differences in the study variables.

Variable	M Mean	F Mean	t	df	*p*	Cohen’s d
Child age	7.184	7.585	−1.432	153.857	0.154	−0.228
Parent age	40.421	41.488	−1.246	135.412	0.215	−0.201
Parent resilience	28.421	29.488	−1.372	144.885	0.172	−0.220
Child resilience	29.868	30.000	−0.275	152.277	0.784	−0.044
Parent flooding	57.421	58.146	−0.359	149.853	0.720	−0.057
Total difficulties SDQ	10.355	9.707	0.672	148.771	0.502	0.108
Novelty seeking JTCI	8.197	7.476	1.225	155.954	0.222	0.194
Harm avoidance JTCI	9.013	9.268	−0.362	155.482	0.718	−0.058
Reward dependence JTCI	5.855	6.085	−0.697	152.471	0.487	−0.111
Persistence JTCI	2.566	2.671	−0.385	155.371	0.701	−0.061
ACE	0.592	0.561	0.156	145.434	0.877	0.025
COVID-19 risk index	0.43	0.37	0.692	152.906	0.490	0.110

Note. The Welch approximation to the degrees of freedom is used. SDQ = Strengths and Difficulties Questionnaire; JTCI = Junior Temperament and Character Inventory; ACE = Adverse Childhood Experiences.

**Table 2:** Correlations among the study variables.

	2	3	4	5	6	7	8	9	10	11	12
1. Child age	0.20 *	−0.04	−0.06	0.06	−0.12	−0.10	−0.02	0.05	−0.04	0.11	0.031
2. Parent age	-	−0.03	0.03	0.06	−0.15	−0.10	0.01	0.04	−0.08	−0.05	0.061
3. Parent resilience		-	0.49 ***	0.27 ***	−0.38 ***	−0.20 *	−0.13	0.20 *	0.18 *	−0.17 *	−0.065
4. Child resilience			-	0.28 ***	−0.58 ***	−0.38 ***	−0.15	0.31 ***	0.41 ***	−0.33 ***	−0.128
5. Parent flooding				-	−0.47 ***	−0.18 *	0.04	0.27 ***	0.12	−0.20 *	−0.092
6. Total difficulties SDQ					-	0.54 ***	0.17 *	−0.24 **	−0.44 ***	0.41 ***	0.091
7. Novelty seeking JTCI						-	−0.29 ***	−0.24 **	−0.45 ***	0.24 **	−0.038
8. Harm avoidance JTCI							-	−0.08	0.02	0.05	0.093
9. Reward dependence JTCI								-	0.02	−0.20 *	−0.057
10. Persistence JTCI									-	−0.26 **	0.012
11. ACE										-	0.188 *
12. COVID-19 risk index											-

Note. Significance levels * *p* < 0.05, ** *p* < 0.01, *** *p* < 0.001. SDQ = Strengths and Difficulties Questionnaire; JTCI = Junior Temperament and Character Inventory; ACE = Adverse Childhood Experiences.

**Table 3:** Hierarchical regression analysis of predictors of total difficulties SDQ (whole sample).

Predictor Variables	Model 1	Model 2	Model 3	Model 4	Model 5	Model 6
Child resilience	−0.579 ***	−0.577 ***	−0.317 ***	−0.286 ***	−0.226 ***	−0.205 **
COVID-19 risk index		0.017	0.045	0.016	0.000	0.001
Novelty seeking JTCI			0.442 ***	0.424 ***	0.419 ***	0.416 ***
Harm avoidance JTCI			0.250 ***	0.244 ***	0.271 ***	0.267 ***
Reward dependence JTCI			−0.012	0.007	0.069	0.070
Persistence JTCI			−0.115	−0.089	−0.089	−0.091
ACE				0.178 **	0.151 **	0.151 **
Parent flooding					−0.318 ***	−0.311 ***
Parent resilience						−0.048
*R* ^2^	0.335	0.336	0.525	0.551	0.639	0.640
*R*^2^ change	0.335	0.000	0.190	0.026	0.087	0.002

Note. Significance levels ** *p* < 0.01, *** *p* < 0.001. SDQ = Strengths and Difficulties Questionnaire; JTCI = Junior Temperament and Character Inventory; ACE = Adverse Childhood Experiences.

**Table 4:** Hierarchical regression analysis of predictors of total difficulties SDQ (boys only).

Predictor Variables	Model 1	Model 2	Model 3	Model 4	Model 5	Model 6
Child resilience	−0.651 ***	−0.646 ***	−0.362 **	−0.357 **	−0.284 **	−0.230 **
COVID-19 risk index		0.021	−0.006	−0.030	−0.026	−0.022
Novelty seeking JTCI			0.464 ***	0.415 **	0.314 **	0.318 **
Harm avoidance JTCI			0.182	0.158	0.148	0.149
Reward dependence JTCI			0.012	0.023	0.112	0.122
Persistence JTCI			−0.085	−0.062	−0.092	−0.102
ACE				0.153	0.129	0.128
Parent flooding					−0.419 ***	−0.399 ***
Parent resilience						−0.090
*R* ^2^	0.423	0.424	0.581	0.598	0.737	0.742
*R*^2^ change	0.423	0.001	0.157	0.018	0.139	0.004

Note. Significance levels ** *p* < 0.01, *** *p* < 0.001. SDQ = Strengths and Difficulties Questionnaire; JTCI = Junior Temperament and Character Inventory; ACE = Adverse Childhood Experiences.

**Table 5:** Hierarchical regression analysis of predictors of total difficulties SDQ (girls only).

Predictor Variables	Model 1	Model 2	Model 3	Model 4	Model 5	Model 6
Child resilience	−0.496 ***	−0.496 ***	−0.276 ***	−0.224 *	−0.186	−0.192
COVID-19 risk index		−0.011	0.043	0.019	0.005	0.005
Novelty seeking JTCI			0.429 ***	0.439 ***	0.465 ***	0.466 ***
Harm avoidance JTCI			0.319 **	0.325 ***	0.358 ***	0.360 ***
Reward dependence JTCI			−0.039	−0.014	0.023	0.024
Persistence JTCI			−0.135	−0.112	−0.106	−0.106
ACE				0.206 *	0.192 *	0.192 *
Parent flooding					−0.216 *	−0.217 *
Parent resilience						0.019
*R* ^2^	0.246	0.246	0.480	0.516	0.557	0.558
*R*^2^ change	0.246	0.000	0.233	0.036	0.042	0.000

Note. Significance levels * *p* < 0.05, ** *p* < 0.01, *** *p* < 0.001. SDQ = Strengths and Difficulties Questionnaire; JTCI = Junior Temperament and Character Inventory; ACE = Adverse Childhood Experiences.

## Data Availability

Data may be obtained from the first author upon reasonable request.
